# Dynamic observations of CRISPR-Cas target recognition and cleavage heterogeneities

**DOI:** 10.1515/nanoph-2022-0286

**Published:** 2022-08-30

**Authors:** Zhijia Zhang, Haechan Jeong, Di Zu, Xintao Zhao, Pramith Senaratne, John Filbin, Brett Silber, Sarah Kang, Ann Gladstone, Matthew Lau, Guangjie Cui, Younggeun Park, Somin Eunice Lee

**Affiliations:** Department of Electrical & Computer Engineering, Biomedical Engineering, Applied Physics, Biointerfaces Institute, Macromolecular Science & Engineering, University of Michigan, Ann Arbor, USA; Department of Mechanical Engineering, University of Michigan, Ann Arbor, USA

**Keywords:** bioplasmonics, Cas, clustered regularly interspaced short palindromic repeats, gene editing, gold nanorod, plasmonics, sgRNA

## Abstract

CRISPR-Cas systems (clustered regularly interspaced short palindromic repeats) have shown great potential as efficient gene editing tools in disease therapeutics. Although numerous CRISPR-Cas systems have been developed, detailed mechanisms of target recognition and DNA cleavage are still unclear. In this work, we dynamically observe the entire process of conjugation, target recognition and DNA cleavage by single particle spectroscopy of CRISPR-Cas systems on single particle surfaces (gold) with the unique advantage of extended time periods. We show the CRISPR-Cas system, comprised of Cas endonuclease and single guide RNA, is stable and functional on single particle surfaces. Owing to the photostability of single particle surfaces, we directly observe in real time the entire dynamic process of conjugation, target recognition and DNA cleavage without photobleaching. We find heterogeneity in target recognition and DNA cleavage processes in which individual spectra vary significantly from one another as well as from the ensemble. We believe an in depth understanding of heterogeneities in CRISPR-Cas systems can overcome potential barriers in precision medicine and personalized disease therapeutics.

## 1 Introduction

CRISPR-Cas systems (clustered regularly interspaced short palindromic repeats) have rapidly emerged as powerful gene editing tools in life science research and disease therapeutics [1], [2], [3]. RNA-guided DNA targeting by CRISPR-associated protein (Cas) enables cleavage at precise sites in the genome by simple complementarity between a single guide RNA (sgRNA) and the target genomic DNA. Despite widespread adoption, large variations in on-target efficiency have been observed across target sites [4]. Many studies [5], [6], [7], [8], [9], [10] indicate that on-target efficiency depends on sgRNA sequence and experimental conditions; however, achieving on-target efficiency still remains challenging. Importantly, studies have shown that, despite being on-target, binding of RNA-guided Cas to the target DNA adjacent to the protospacer adjacent motif does not always result in cleavage, and the target DNA can remain uncleaved [4]. Resolving variations in on-target efficiency will be critically important if CRISPR-Cas are to be widely used in therapeutic applications. As on-target efficiency remains a persistent problem, methods that would allow for dynamic observation of the entire process of target recognition and DNA cleavage over extended time periods could enable a new way to verify gene editing in real time.

To study target recognition and cleavage, state-of-the-art single molecule fluorescence imaging [11, 12] has allowed for probing real-time interactions between fluorescently labeled Cas/sgRNA and fluorescently labeled target DNA. However, state-of-the-art fluorescence methods are limited by photobleaching and cannot observe events over extended time periods. Single particle spectroscopy methods [13], [14], [15], [16] have observed events without photobleaching, but have not been applied to follow CRISPR-Cas target recognition and cleavage processes in real time. Highspeed atomic force microscopy [17] has visualized Cas structural dynamics without photobleaching. As atomic force microscopy methods require direct contact with Cas, intracellular visualization of target recognition and DNA cleavage is not feasible.

**Figure 1:**
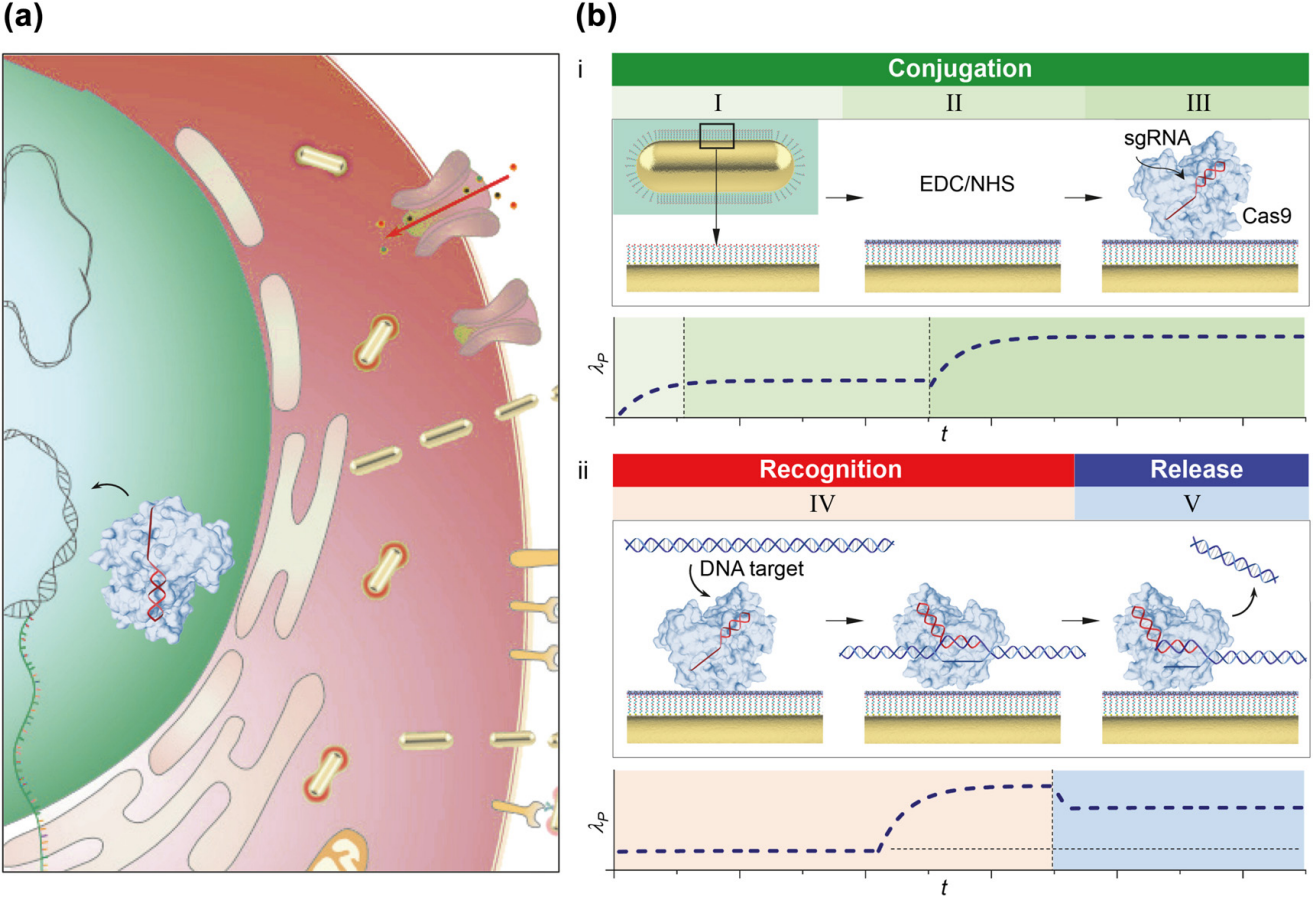
Dynamic observations of CRISPR-Cas target recognition and cleavage. (a) Conceptual schematic of RNA-guided DNA targeting by CRISPR-associated protein (Cas) enables cleavage at precise sites in the genome by simple complementarity between a single guide RNA (sgRNA) and the target genomic DNA in cells. (b i) Conceptual schematic of dynamic observations of CRISPR-Cas conjugation. CRISPR-Cas system, comprised of Cas9 and sgRNA, is conjugated on a single particle surface (Bio-AuNRs) immobilized on a self-assembled monolayer. Conceptual schematic of single particle scattering peak wavelength *λ*_P_ versus time *t* during a three-stage conjugation process of priming (I), EDC/NHS conjugation (II) and CRISPR-Cas conjugation (III). (b ii) Conceptual schematic of dynamic observations of CRISPR-Cas target recognition and cleavage. Conceptual schematic of single particle scattering peak wavelength *λ*_P_ versus time *t* of target recognition of short purified DNA targets (IV) and release of DNA products (V).

Here, we utilize single particle spectroscopy of CRISPR-Cas systems on single particle surfaces (gold) to dynamically observe the entire process of conjugation, target recognition and cleavage with the unique advantage of extended time periods. We found the CRISPR-Cas system, comprised of Cas9 bound with sgRNA, is stable and functional on single particle surfaces as compared to Cas9 alone. We directly observed in real time the entire dynamic process of CRISPR-Cas conjugation, target recognition and cleavage without photobleaching owing to the photostability of single particle surfaces. We found heterogeneity in target recognition and cleavage processes in which individual spectra vary significantly from one another and deviate significantly from the ensemble with profound implications on on-target efficiencies. In the future, we envision heterogeneity learned by the single-particle spectroscopy will provide real time feedback to guide CRISPR-Cas target recognition and cleaving processes (Figure 1) towards precision medicine and personalized disease therapeutics.

## 2 Results and discussion

**Figure 2:**
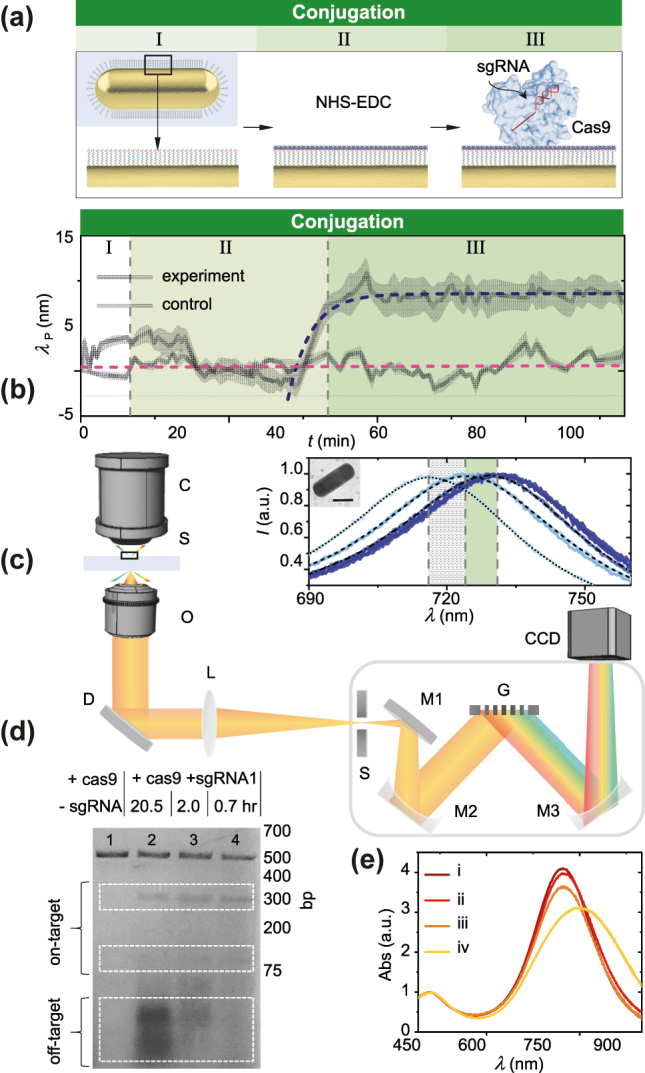
CRISPR-Cas conjugation process is dynamically observed on single particles surfaces. (a) Conceptual schematic of conjugation process of priming (I), EDC/NHS conjugation (II) and CRISPR-Cas conjugation (III) on single particle surface (BioAuNR). (b) Single particle scattering peak wavelength *λ*_P_ versus time *t* for negative control in buffer only (pink color) and experiment (blue color). Line: averaged spectrum, Shading: spectrum envelope. (c) Experimental setup consisting of transmission darkfield microscope outfitted with spectrometer; C: condenser; S: sample; O: objective; D: dichroic mirror; L: lens; S: slit; M1, M2, M3: mirrors; G: grating. Representative scattering spectra at each stage in the conjugation process. Inset: transmission electron microscopy (TEM) image. Scale bar: 35 nm. (d) Bulk agarose gel electrophoresis. Lane 1: negative control: apo Cas9 (Cas9 without sgRNA); Lane 2: Cas9 complexed with sgRNA that was synthesized with incubation time of 20.5 h; Lane 3: Cas9 complexed with sgRNA that was synthesized with incubation time of 2.0 h; Lane 4: Cas9 complexed with sgRNA that was synthesized with incubation time of 0.7 h. (e) Bulk UV–vis absorbance spectra during bulk BioAuNR conjugation process. i: BioAuNRs in buffer only; ii: EDC/NHS conjugation of BioAuNRs following step i; iii: Conjugation of BioAuNRs to Cas9 complexed with sgRNA1 following step ii; iv. Conjugation of BioAuNRs to apo Cas9 following step ii.

In this study, we asked the question: do CRISPR-Cas dynamics exhibit heterogeneities which deviate significantly from the ensemble? To answer this question, light scattering provides a means to observe the dynamic process of CRISPR-Cas target recognition and cleavage over time. In single particle spectroscopy, the scattering signal is proportional to the polarizability which depends on the permittivity of the local environment [18]. Binding of RNA-guided Cas to the target DNA and subsequent cleavage of the target DNA taking place on the single particle surface changes the permittivity of the local environment and thus the scattering signal. Using a dark-field microscope outfitted with a spectrometer (Figure 2), we continuously acquired time course single particle scattering spectra by dispersing the wavelengths of scattered light using a diffraction grating and obtaining scattering intensity as a function of wavelength during the entire process of CRISPR-Cas conjugation, target recognition and cleavage.

We firstly generated a set of 3 sgRNAs (herein referred to as sgRNA1, sgRNA2 and sgRNA3) targeting multiple sites within human amyloid beta precursor protein (APP) loci (Figure S1). APP is a transmembrane protein, that when cleaved by β-secretases and γ-secretases, leads to Aβ production in Alzheimer’s disease. To validate that CRISPR-Cas could selectively cleave target DNA, we assessed on-target and off-target cleavage using short purified DNA targets of APP from neuroblastoma (SH-SY5Y) cells. We chose SH-SY5Y for its potential use as an *in vitro* model of Alzheimer’s disease. We compared Cas9 complexed with either sgRNA1, sgRNA2 or sgRNA3 against apo Cas9 (Cas9 without sgRNA). On-target and off-target cleavage were optimized by titrating the amount of Cas9 and sgRNA. Agarose gel electrophoresis (Figure 2(d), S2) showed that Cas9 complexed with sgRNA1 (Figure 2(d)) resulted in the highest on-target cleavage with the lowest off-target cleavage as compared to Cas9 complexed with sgRNA2 (Figure S2a) or Cas9 complexed with sgRNA3 (Figure S2b). These results validate that CRISPR-Cas can effectively cleave DNA targets into shorter fragments.

Having determined sgRNA1 is effective against the target, we conjugated CRISPR-Cas to single particle surfaces. We chose plasmonic gold nanorods [19], [20], [21], [22], [23], [24], [25], [26], [27], [28], [29], [30], [31], [32], [33] because of their strong and stable scattering properties owing to the collective oscillation of electron density at the metal-dielectric interface. We synthesized biocompatible plasmonic gold nanorods (Bio-AuNRs) by utilizing a bromide-free surfactant CTAC to achieve CTA^+^ free [34]. Ligand exchange was conducted to replace CTAC groups with methoxy polyethylene glycol (m-PEG) and carboxyl polyethylene glycol (PEG-COOH) groups. EDC/NHS (1-Ethyl-3-(3-dimethylaminopropyl) carbodiimide/N-hydroxysulfosuccinimide) activation of carboxyl groups on single particle surfaces was conducted to conjugate to free amine groups on Cas9. Using bulk UV–Vis spectroscopy, we compared the stability of Cas9 complexed with sgRNA1 against apo Cas9 (Cas9 without sgRNA) during the conjugation process (Figure 2(e)). After removal of unbound molecules via centrifugation and resuspension, Cas9 complexed with sgRNA1 showed a peak redshift (2 nm) with no broadening of the peak bandwidth, suggesting stable conjugation with Bio-AuNRs. We attributed peak decrease to the removal/resuspension process. In contrast, Bio-AuNRs conjugated with apo Cas9 were unstable and readily aggregated (Figure S3). These findings indicate that conjugation was unstable with apo Cas9 whereas conjugation was stable with Cas9 complexed with sgRNA (Figure 2(e), S3). These findings are important because they demonstrate that Cas9 must be complexed first with sgRNA and then conjugated to single particle surfaces to ensure stability. As crystallography studies [35] have shown that Cas9 wraps around sgRNA to form a tightly bound structure, we reasoned that Cas9 complexed with sgRNA may provide structural stability to facilitate the conjugation process. After bulk analysis, we then proceeded with real time observation of the conjugation process at the single particle level. We used surface-immobilized Bio-AuNRs as anchors to a self-assembled monolayer. The carboxyl groups on the Bio-AuNRs allowed for binding to an amine-terminated self-assembled monolayer. Following immobilization of the anchors, we continuously acquired time course single particle scattering spectra during a three-stage conjugation process (Figure 2(b)): In stage I, we firstly buffer exchanged the chamber in order to prime the chamber. At higher ionic strength, we expect more ions to shield the charged surface, altering the permittivity of the local environment. We observed a peak redshift (8 nm) when DI water was exchanged with buffer. In stage II, we introduced EDC/NHS into the chamber to activate carboxyl groups on single particle surfaces. In stage III, we then introduced CRISPR-Cas into the chamber to conjugate free amine groups on Cas9 with carboxyl groups on single particle surfaces. Binding of CRISPR-Cas to single particle surfaces resulted in a peak redshift (7 nm). Binding of CRISPR-Cas of various aspect ratio BioAuNRs resulted in a similar peak redshift (Figure S4). No significant peak shift was observed in the unconjugated control flushed with buffer.

**Figure 3:**
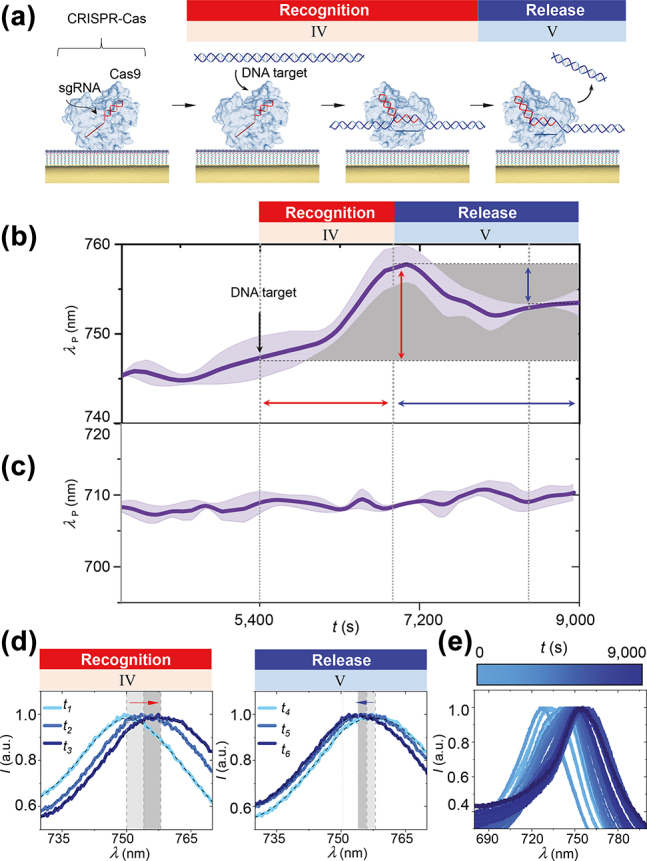
CRISPR-Cas target recognition and cleavage process is dynamically observed on single particle surfaces. (a) Conceptual schematic of target recognition and cleavage process. (b) Single particle scattering peak wavelength *λ*_P_ versus time *t* introducing DNA target at *t* = 5400 s. Line: averaged spectrum, Shading: spectrum envelope. (c) Single particle scattering peak wavelength *λ*_P_ versus time *t* for negative control in buffer only. (d) Representative scattering spectra at various times during the target recognition process. *t*_1_ = 5700 s; *t*_2_ = 6300 s; *t*_3_ = 6900 s. Representative scattering spectra at various times during the cleavage process. *t*_4_ = 7200 s; *t*_5_ = 7800 s; *t*_6_ = 8400 s. (e) Time course scattering spectra during target recognition and cleavage process.

With CRISPR-Cas conjugated to single particle surfaces, we investigated target recognition and cleavage processes in real time. The entire process of CRISPR-Cas conjugation, target recognition and cleavage was a five-stage process (Figure S5). Conjugation took place in stages I-III. We confirmed conjugation of CRISPR-Cas to single particle surfaces by the peak redshift in stage III which then plateaued, indicating conjugation was stabilized within 2000 s. We posited that binding and release of charged species, DNA targets, should modify the permittivity of the local environment. Upon introduction of short purified DNA targets into the chamber in stage IV, we firstly observed a gradually rising plateau over 1000 s that we attributed to diffusion of the DNA targets to CRISPR-Cas and target searching. We then detected target recognition by the appearance of a peak redshift (8 nm) from CRISPR-Cas binding with target DNA (Figure 3). Shortly after, this binding event was then followed by a partial peak blueshift (4 nm) in stage V indicating a cleaving event. We observed the partial peak blueshift occurred over approximately 1000 s and then plateaued. As the cleaving event produced two cleaved products, the partial peak blueshift suggested CRISPR-Cas released one of the cleaved products and the following plateau indicated CRISPR-Cas remained tightly bound to other cleaved products. No significant peak shift was observed in the absence of target DNA.

**Figure 4:**
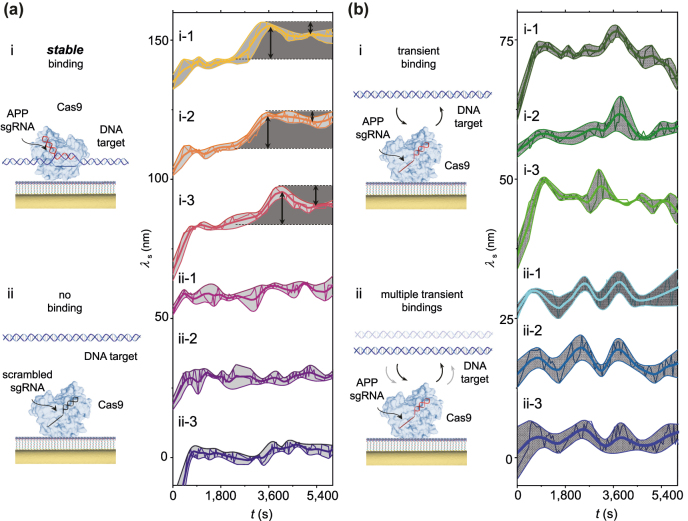
Dynamic observations of CRISPR-Cas target recognition and cleavage heterogeneities. (a i) Conceptual schematic of stable binding of target DNA with CRISPR-Cas complexed with APP sgRNA. Stable binding i-1, i-2, i-3: Single particle wavelength shift *λ*_s_ versus time *t* showing stable binding and cleavage. Shading (dark gray): Stable binding events with target DNA consistently resulted in a significant peak redshift and cleaving events consistently resulted in a partial peak blueshift. Thin line: raw spectrum, Thick line: averaged spectrum, Shading (light gray): spectrum envelope. (a ii) Conceptual schematic of no binding of target DNA with CRISPR-Cas complexed with scrambled sgRNA. Scrambled sgRNA ii-1, ii-2, ii-3: Single particle wavelength shift *λ*_s_ versus time *t*. Thin line: raw spectrum, Thick line: averaged spectrum, Shading (light gray): Spectrum envelope. (b i) Conceptual schematic of transient binding of target DNA with CRISPR-Cas complexed with APP sgRNA. Transient binding i-1, i-2, i-3: Single particle wavelength shift *λ*_s_ versus time *t*. Thin line: raw spectrum, Thick line: averaged spectrum, Shading (light gray): spectrum envelope. (b ii) Conceptual schematic of multiple transient bindings of target DNA with CRISPR-Cas complexed with APP sgRNA. Multiple transient bindings ii-1, ii-2, ii-3: Single particle wavelength shift *λ*_s_ versus time *t*. Thin line: raw spectrum, Thick line: averaged spectrum, Shading (light gray): spectrum envelope.

Finally, we investigated variations in gene editing. Binding of CRISPR-Cas to the target DNA does not always result in cleavage [4]. Dynamic observation of the entire process of target recognition and DNA cleavage enabled real time verification of gene editing. We studied multiple CRISPR-Cas conjugated to single particle surfaces (Figure 4). We found individual spectra vary significantly from one another as well as from the ensemble. We verified that stable binding events with target DNA consistently resulted in a significant peak redshift and cleaving events consistently resulted in a partial peak blueshift (Figure 4(a i)). In addition to stable binding, we observed transient binding of target DNA which did not result in cleavage (Figure 4(b i)). Upon introduction of target DNA, CRISPR-Cas attempted to bind target DNA. We detected a peak redshift followed by a peak blueshift returning back to the baseline, indicating transient binding followed by unbinding that did not result in cleavage. We also observed repeated (multiple) attempts of CRISPR-Cas to bind target DNA (Figure 4(b ii)). We detected repeated peak redshifts and blueshifts back to the baseline, indicating multiple attempts which did not result in cleavage. Additionally, we calculated the average of the individual spectra (Figure S6). Cleavage and release, evident by the partial peak blueshift in individual spectra (Figure 4(a i)), was not clearly visible in the ensemble average spectra. These findings suggest that CRISPR-Cas dynamics can be hidden by ensemble averaging and highlight a need for individual analysis. No significant peak shifts were observed when target DNA was introduced to Cas9 complexed with scrambled sgRNA comprised of a random sequence (Figure 4(a ii)). Heterogeneity in individual spectra show the occurrence of both stable and unstable binding of CRISPR-Cas and also demonstrate cleavage and release only occurred following stable binding. Variations in individual spectra highlight the potential for real time verification of gene editing to improve therapeutic outcomes.

## 3 Conclusions

In conclusion, we have dynamically observed the entire process of conjugation, target recognition and DNA cleavage by single particle spectroscopy of CRISPR-Cas systems on single particle surfaces. We have shown heterogeneity in target recognition and DNA cleavage processes in which individual spectra vary significantly from one another as well as from the ensemble. Real-time verification of gene editing may aid in resolving on-target efficiencies for therapeutic applications. For example, precise delivery of CRISPR-Cas systems to a desired intracellular site (Figure 1(a)) can be achieved by optical imaging with manipulation. At the desired site, we envision real-time verification of gene editing. A current limitation is the efficient and specific delivery of CRISPR-Cas. This can be overcome using optical manipulation methods, such as optical trapping, for controlled manipulation of CRISPR-Cas. This method is compatible with various nanoparticle geometries (spheres, rods, etc.). We anticipate real time verification with precise delivery will improve the likelihood of achieving a desired gene editing result and will improve therapeutic outcomes.

## Supplementary Material

Supplementary Material Details
