# Supplementary material for: Dynamic observations of CRISPR-Cas target recognition and cleavage heterogeneities
Source: Nanophotonics. 2022 Aug 30;11(19):4419–25. doi: 10.1515/nanoph-2022-0286 (PMC11502048; doi:10.1515/nanoph-2022-0286)
Supplement: Supplementary file 1 — Supplementary Material Details [file j_nanoph-2022-0286_suppl.pdf]

Supporting information

Dynamic observations of CRISPR-Cas target recognition and cleavage heterogeneities

*Zhijia Zhang[1], Haechan Jeong[1], Di Zu[1], Xintao Zhao[1], Pramith Senaratne[1], John Filbin[1], Brett Silber[1], Sarah Kang[1], Ann Gladstone[1], Matthew Lau[1], Guangjie Cui[1], Younggeun Park[2], Somin Eunice Lee[1]**

[1]Department of Electrical & Computer Engineering, Biomedical Engineering, Applied Physics, Biointerfaces Institute, Macromolecular Science & Engineering, University of Michigan, [2]Department of Mechanical Engineering, University of Michigan

* To whom correspondence should be addressed.

Materials

Hexadecyltrimethylammonium chloride (CTAC), hexadecyltrimethylammonium bromide (CTAB), sodium oleate (NaOL), hydrogen tetrachoooroaurate (III) trihydrate ($HAuCl_4 \cdot 3H_2O$), acetone, calcium chloride ($CaCl_2$), 2-propanol (IPA), l-ascorbic acid, silver nitrate ($AgNO_3$), sodium borohydride ($NaBH_4$), hydrochloric acid (HCl), 6-mercaptohexanoic acid (MHA), 1-dodecanethiol (DDT), toluene, methanol, sodium chloride (NaCl), methoxy polyethylene glycol thiol(m-PEG), poly(ethylene glycol) 2-mercaptoethyl ether acetic acid(PEG-COOH), 2-(N-morpholino) ethanesulfonic acid monohydrate (MES), N-(3-dimethylaminopropyl)-N′-ethylcarbodiimide hydrochloride (EDC), N-hydroxysulfosuccinimide sodium salt (Sulfo-NHS), phosphate buffered saline (PBS), ethylenediaminetetraacetic acid (EDTA), 3-Aminopropyl)triethoxysilane (APTES), sodium acetate, methylene, and ethanol were purchased from Sigma-Aldrich. Dulbecco's modified eagle medium (DMEM), 1X tris-borate-EDTA buffer (TBE), proteinase K, trypsin-Ethylenediaminetetraacetic acid (EDTA), fetal bovine serum (FBS), and phosphate buffered saline (PBS) were purchased from Thermo Fisher Scientific. DNeasy kit was purchased from Qiagen. PCR Mastermix, PCR primers, and protein degrader were purchased from Applied Biological Materials. Single guide RNA (sgRNA) synthesis kit, Cas9 protein, and Cas9 reaction buffer were purchased from New England Biolabs. Human neuroblastoma cell line (SH-SY5Y) was purchased from the American Type Culture Collection (ATCC).

Methods

Sample Preparation

Cell Culture & Lysis

SH-SY5Y cells were cultured in media supplemented with 10% FBS and maintained in a 37°C incubator with 5% CO_2 humidified air. Cells were seeded at 0.4-1.2x10^5 cells per well in a 6-well plate and grown to 1.0x10^6 cells per well. From each well, cells were trypsinized and transferred to a 15 mL centrifuge tube. Cells were resuspended in 200 μL of PBS and centrifuged at 367xg RCF for 3 minutes. Supernatant was aspirated and the remaining cell pellet was resuspended in 50 μL PBS and 3 μL proteinase K in preparation for cell lysis. To the cell suspension, 50 μL cell lysis buffer (AL, DNeasy kit) was added. The cell suspension with cell lysis buffer was then mixed and incubated with agitation on a shaker at 56°C for 10 minutes.

Preparation of Target DNA

DNA extraction was carried out by DNeasy kit following manufacturer instructions. Working solution of wash buffer AW1 and wash buffer AW2 were prepared in ethanol and preheated to 56°C with agitation on a shaker for 10 minutes. To the lysate, 50 μL of ethanol (96-100%) was added and vortexed. The lysate was pipetted into a mini spin column, which was then placed in a 2 mL collection tube and centrifuged at 5,878xg RCF for 1 minute. The flow-through was then discarded, and the spin column was placed in a new 2 mL collection tube. Then 50 μL of wash buffer AW1 was added to the spin column, and the tube was centrifuged at 5,878xg RCF for 1 minute. The flow-through was

discarded and the spin column was placed in a new 2 mL collection tube. Then 50 μL of wash buffer AW2 was added to the spin column, and the tube was centrifuged at 18,001xg RCF for 3 minutes. The flow-through was discarded, and the spin column was placed in a 1.5 mL microcentrifuge collection tube. Finally, 50 μL of elution buffer AE was added, and the tube was centrifuged at 5,878xg RCF for 1 minute. The final flow-through containing extracted DNA was collected and stored at -80°C.

PCR thermocycler (Biorad) was used to perform PCR amplification. PCR reactions were prepared containing 50 μL 2X PCR Mastermix, 16 μL of extracted DNA, 1 μL forward primer, and 1 μL reverse primer. The reaction volume was supplemented with DI water to reach a total volume of 100 μL. The following program was used to perform PCR reaction (40 cycles) using the PCR thermocycler: 98°C for 120s; 40 cycles: 98°C for 10s, 60°C for 30s, 72°C for 25s; 72°C for 120s. The PCR product was then purified and concentrated by ethanol precipitation.

To purify and concentrate the PCR product, 1.5 μL of sodium acetate was added to 15 μL of PCR product. Then, 60 μL of cold 100% ethanol was added, vortexed and incubated for 15 minutes. Following incubation, the mixture was centrifuged at 13,225xg RCF for 10 minutes. The supernatant was discarded and 200 μL of cold 100% ethanol was added. The mixture was centrifuged at 13,225xg RCF for 10 minutes. The supernatant was discarded and the pellet was air dried. The pellet was resuspended in 20 μL of 10 mM Tris (pH 8.5) to obtain the final purified target DNA product.

Preparation of sgRNA

Synthesis of a set of 3 sgRNAs (sgRNA1, sgRNA2 and sgRNA3) targeting multiple sites within human amyloid beta (APP) loci was carried out using a sgRNA synthesis kit following manufacturer instructions. Reaction containing 4.6 µL nuclease free water, 10 µL sgRNA synthesis Supermix, 1 µL target DNA, 0.4 µL DNA polymerase, and 0.4 µL Onescribe enzyme mix was prepared and briefly centrifuged to mix. The sgRNA was synthesized for different incubation times (0.7 hr, 2.0 hr, 20.5 hr) at 37°C resulting in more sgRNA product with longer incubation time. The sgRNA products were stored at -80°C.

CRISPR-Cas System

To prepare Cas9 protein complexed with sgRNA, 2 µL Cas9 protein (1 pmol/ µL), 1 µL sgRNA (~1 µg), and 2 µL Cas9 reaction buffer were mixed and incubated at 37°C for 15 minutes. To prepare apo Cas9 (Cas9 without sgRNA), 2 µL Cas9 protein (1 pmol/ µL), 1 µL DI water, and 2 µL Cas9 reaction buffer were mixed and incubated at 37°C for 15 minutes.

Agarose Gel Electrophoresis

To compare on-target efficiency by agarose gel electrophoresis, Cas9 was complexed with either sgRNA1 synthesized for 0.7 hr, 2.0 hr, 20.5 hr incubation times, sgRNA2 synthesized for 0.7 hr, 2.0 hr, 20.5 hr incubation times, or sgRNA3 synthesized for 0.7 hr, 2.0 hr, 20.5 hr incubation times. For each condition, 5 µL of Cas9 protein complexed with

sgRNA, 10 µL of purified target DNA, and 5 µL of DI water were mixed and incubated overnight. For the negative control, 5 µL of apo Cas9 protein, 10 µL of purified target DNA, and 5 µL of DI water were mixed and incubated overnight. The following day, 2 µL of protein degrader was added to each sample. Agarose gel (2%(w/v) was prepared with 1X TBE buffer. Samples were loaded into the wells of the agarose gel and separated by electrophoresis for 60 minutes at 100 V in 1X TBE buffer. The gel then was stained using 0.1%(w/v) methylene solution and was de-stained in DI water overnight.

Preparation of CRISPR-Cas-Gold Nanorods

Gold nanorods (27 nm x 75 nm; 36 nm x 96 nm) were synthesized as previously described[1]. CTAC–AuNRs were synthesized by bromide-free seed mediated growth. CTAC was replaced with PEG by round trip phase transfer, forming BioAuNRs. For bulk analysis, EDC/NHS (ratio 3:3.6) was added to samples and incubated for 20 minutes at room temperature. The samples were then centrifuged at 3,306xg RCF for 15 minutes and resuspended in DI water three times. Cas9 protein complexed with sgRNA or apo Cas9 was added to the samples and incubated for 1 hour. The samples were then centrifuged at 6,000 rpm for 15 minutes and resuspended in DI water three times. The samples were then characterized by bulk UV-Vis absorption spectroscopy.

Experimental Setup

Imaging

For real time analysis, a custom flow chamber was constructed. Self-assembled monolayer was prepared on a glass surface. Glass coverslips were cleaned with acetone, IPA and DI water and then baked in an oven at 60℃ for 12 hours to remove water. Subsequently, glass cover slips were plasma treated for 30 seconds before immersing in 350 μL APTES in 45 mL 100% ethanol. After incubation for 24 hours, glass coverslips were sonicated for 30 seconds in ethanol solution and washed 3 times with ethanol to remove excessive APTES. Then the glass coverslips were promptly baked at 120˚C for 3 hours. After baking, glass cover slips were immersed in gold nanorod solution with a resonance wavelength of 695 nm (2 μL OD 50 in 40 mL DI water). After incubation for 12 hours, glass cover slips were washed with DI water and air dried. Flow chamber was then assembled using the modified glass coverslip together with another clean glass coverslip. The flow chamber was configured on a darkfield microscope (Olympus) outfitted with a halogen light source (Olympus), a wet condenser (Olympus), 40x-100x objectives (Olympus), and imaging spectrometer (Princeton Instruments) with a CCD (Princeton Instruments).

Single Particle Analysis

For real time analysis of conjugation, target recognition and cleavage, 5 μM EDC/s-NHS was flowed into the chamber, incubated for 20 minutes, and then rinsed by flowing DI water 3 times. Then 80 μL of Cas9 complexed with sgRNA, Cas9 complexed with

scrambled sgRNA, or apo Cas9 in Cas9 reaction buffer was introduced into the chamber, incubated for 1 hour, and then rinsed by flowing Cas9 reaction buffer 3 times. After conjugation, 80 μL of target DNA was flowed into the chamber, incubated for 45 minutes, and then rinsed with MES buffer 3 times. Single particle spectra were obtained throughout the entire process of conjugation, target recognition and cleavage.

To determine the peak wavelength, we firstly normalized and aligned the baseline of the spectrum. The peak wavelength was determined by finding the maximum value of the spectrum. This value was compared to the absorption maximum of the longitudinal plasmon resonance calculated as a function of aspect ratio and permittivity[2].

Supplementary Figures

Gene	Name	sequence (5' to 3')
APP	DNA oligo: sgRNA1	G T G A T T C C C T A C C G C T G C T T A G G
APP	DNA oligo: sgRNA2	C T G G C A C A C C G T C G C C A A A G A G G
APP	DNA oligo: sgRNA3	G C G G A A T T G A C A A G T T C C G A G G G

Figure S1. Target specific DNA oligos for synthesis of sgRNAs.

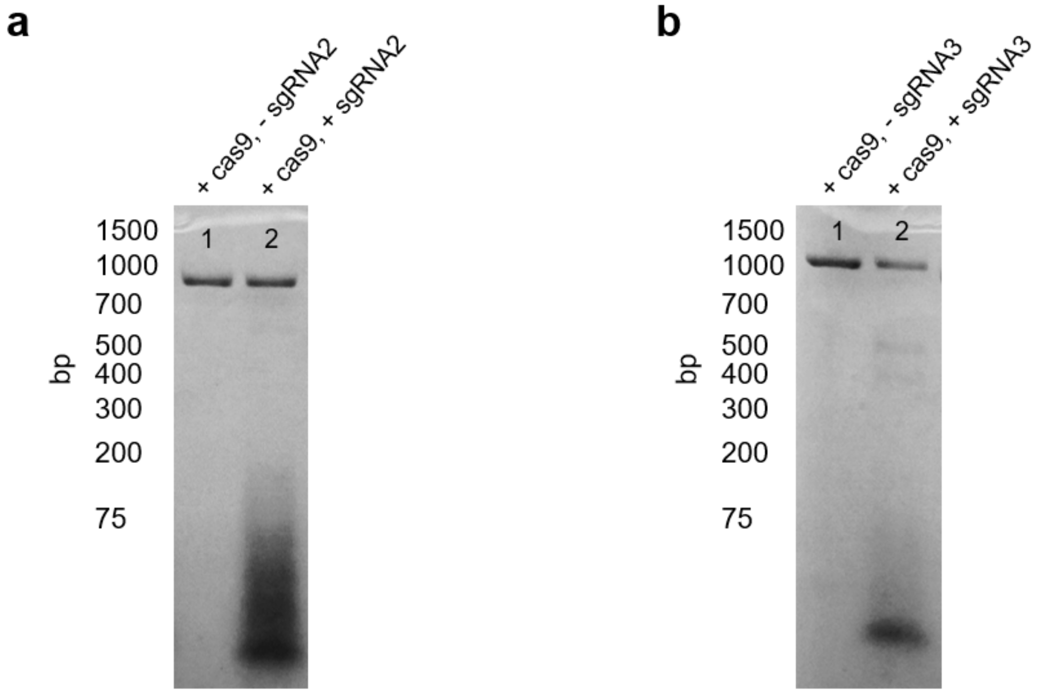

Figure S2. Bulk agarose gel electrophoresis. (a) sgRNA2: Lane 1: negative control: apo Cas9 (Cas9 without sgRNA); Lane 2: Cas9 complexed with sgRNA2 that was synthesized with incubation time of 20.5 hr. **(b) sgRNA3:** Lane 1: negative control: apo Cas9 (Cas9 without sgRNA); Lane 2: Cas9 complexed with sgRNA3 that was synthesized with incubation time of 20.5 hr.

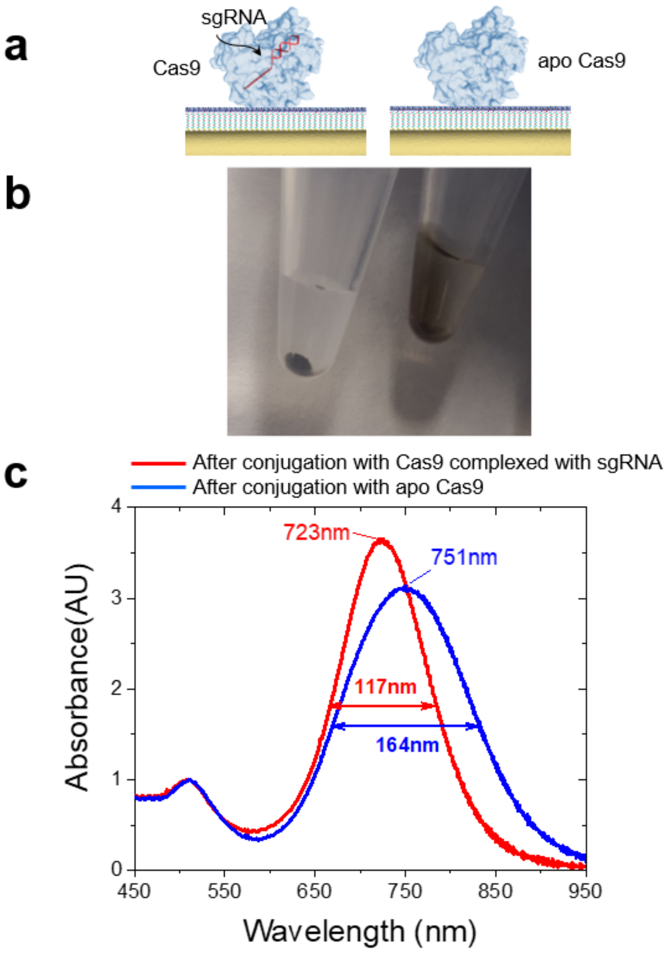

Figure S3. Bulk UV-Vis spectroscopy. (a) Conceptual schematic of Cas9 complexed with sgRNA (left) and apo Cas9 (right). **(b)** Photograph of gold nanorods conjugated with Cas9 complexed with sgRNA (left). Gold nanorods conjugated with apo Cas9 (right) aggregated during conjugation. **(c)** UV-Vis absorbance spectra of gold nanorods conjugated with Cas9 complexed with sgRNA (red color). Gold nanorods conjugated with apo Cas9 aggregated (blue color), resulting in a broadened peak bandwidth and lowered peak intensity.

a

	Diameter (nm)	Length (nm)
	27	75
	36	96

b **c**

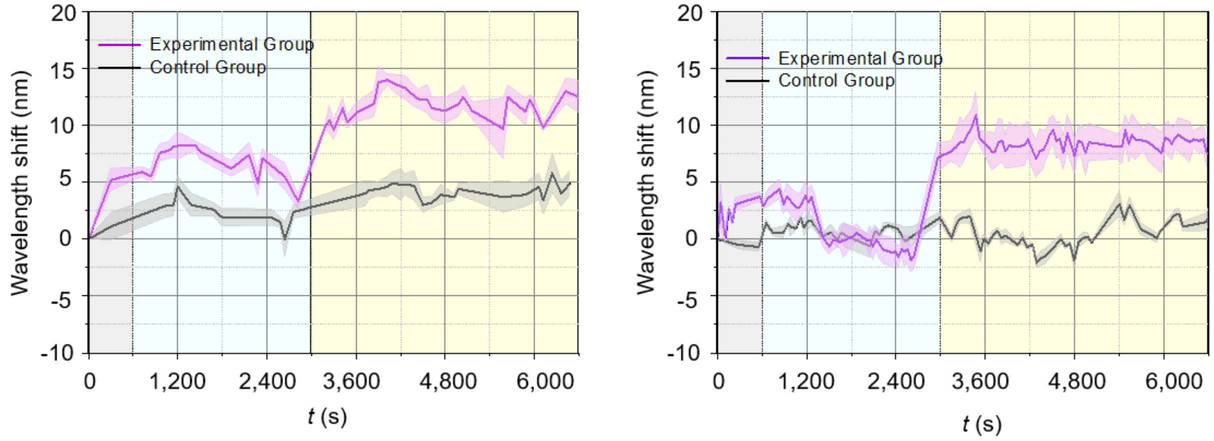

Figure S4. Real-time analysis of conjugation of CRISPR-Cas to different aspect ratio BioAuNRs. (a) Table of geometrical dimensions. **(b)** Single particle wavelength shift versus time *t* for 27 nm x 75 nm BioAuNR. Experiment (purple color): Conjugation of BioAuNR with Cas9 complexed with sgRNA. Control (black color): unconjugated control flushed with buffer only. **(c)** Single particle wavelength shift versus time *t* for 36 nm x 96 nm BioAuNR. Experiment (purple color): Conjugation of BioAuNR with Cas9 complexed with sgRNA. Control (black color): unconjugated control flushed with buffer only.

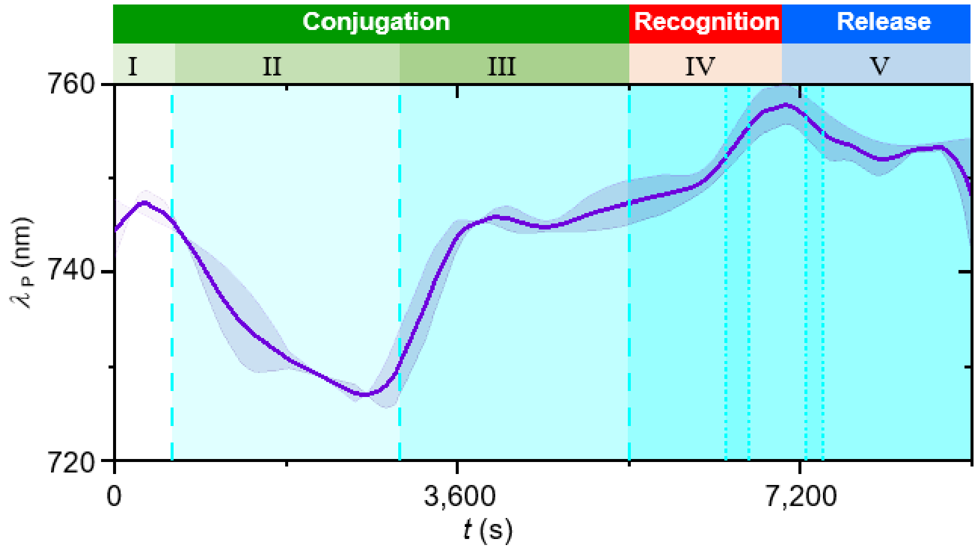

Figure S5. Five stage process of conjugation, target recognition, and cleavage. Single particle scattering peak wavelength λ_P versus time t.

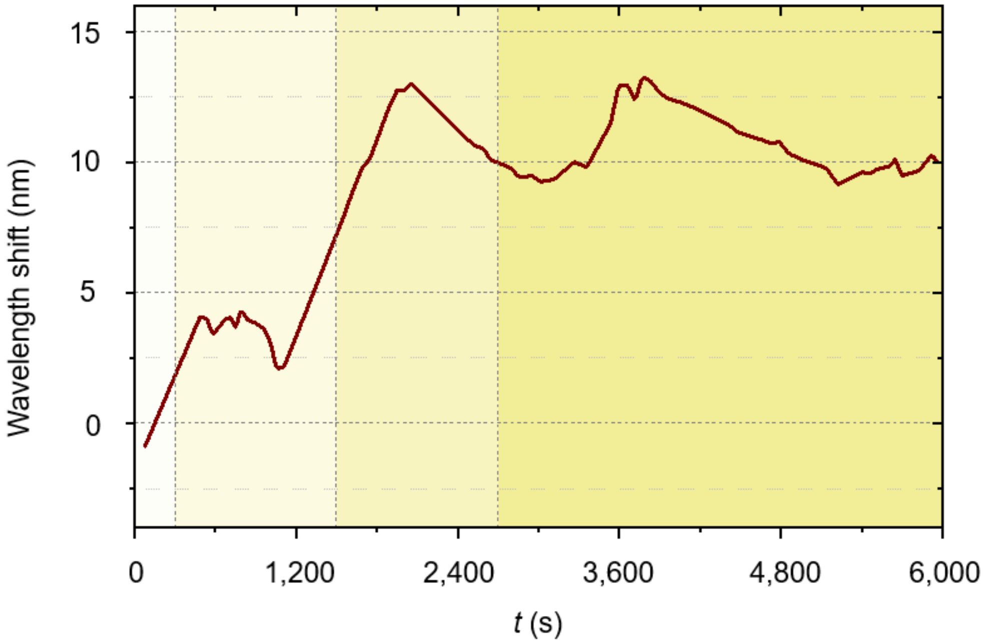

Figure S6. Ensemble average of individual spectra. Ensemble average wavelength shift versus time *t*.

Supplementary References

1. Lin, W. K. *et al.* Optically and Structurally Stabilized Plasmo-Bio Interlinking Networks. *Adv. Mater. Interfaces* **8**, 1–9 (2021).

2. Link, S. & El-Sayed, M. A. Simulation of the Optical Absorption Spectra of Gold Nanorods as a Function of Their Aspect Ratio and the Effect of the Medium Dielectric Constant. *J. Phys. Chem. B* **109**, 10531–10532 (2005).
